# Frequency of psychosocial stress and its relationship to safety attitude towards nurse’s performance at tertiary care hospitals

**DOI:** 10.12669/pjms.40.5.7913

**Published:** 2024

**Authors:** Abdul Wahid, Badil, Washdev, Sabir Hussian

**Affiliations:** 1 Abdul Wahid, Benazir College of Nursing, Shaheed Mohtarma Benazir Bhutto, Medical University, Larkana, Pakistan; 2 Badil, Dow Institute of Nursing and Midwifery, Dow University of Health Sciences, Karachi, Pakistan; 3 Washdev, Psychiatric Department, Dr. Ruth KM Pfau Civil Hospital, Karachi, Pakistan; 4 Sabir Hussian, Benazir College of Nursing, Shaheed Mohtarma Benazir Bhutto, Medical University, Larkana, Pakistan

**Keywords:** Psychosocial Stress, Safety attitude, Performance, Nurses, Tertiary Care Hospital

## Abstract

**Background & Objectives:**

Psychosocial stress has a detrimental effect on nurses’ work performance. A safe working environment is significant in providing nurses with safe and satisfactory care. The objective of study was to assess the frequency of psychosocial stress of nurses and determine the relationship between psychosocial stress of nurses and safety attitude towards nurses’ performances at Tertiary Care Hospital, Karachi.

**Methods:**

Analytical cross-sectional study was conducted at Dr. Ruth KM Pfau Civil Hospital, Karachi, and Dow University Hospital Karachi for six months, from December 2020 to May 2021.

A total 260 participants were approached by a non-probability purposive sampling. Pearson’s correlation was used to establish the relationship between the psychosocial stress of nurses and different parameters of their safety attitude. The Chi-square test was applied for the association between demographic factors of nurses with their psychosocial stress levels. A p-value of ≤0.05 was considered as significant.

**Results:**

The majority of nurses, 180 (69.2%), described poor health, while 54 (20.8%) had good health, and only 10% (26) of nurses reported their best health status. Three parameters were negatively correlated and statistically significant with psychosocial stress, namely: teamwork (r-0.13<0.002), job satisfaction (r-0.15<0.028), and perception of management (r-0.34<0.000). The result of the study indicated that gender (P-value<0.000), marital status (P-value<0.0037), and institution (P-value <0.005) were significantly associated with safety attitude score.

**Conclusion:**

Most of the nurses had poor health, which was significantly related to teamwork, job satisfaction and perception of management, and stress recognition.

## INTRODUCTION

Nurses’ role is highly significant in providing safe and satisfactory care; it depends on their working environment apart from their proficiency, knowledge, competency, and skills.^1^ Health care providers are more prone to face social and mental issues, and nurses rank on top of the list in the health care system.^2^ These factors include lack of support, unsafe environment, poor working conditions of nursing staff, lack of resources, and poor participation in decision-making.^3^

Furthermore, psychosocial health is one of the crucial elements of the physical health of nurses, which is directly affected by the organizational environment of the healthcare system.^4^ Because of the more substantial workload, nursing staff did not have sufficient time to complete their assigned tasks appropriately, consequently, contributing to many adverse outcomes in the workplace 5, including unsafe patient handling in mobilization and transferring, poor drug labeling, pressure ulcer, and hospital-acquired infections.^6^

Promoting the psychosocial health of nurses is an alarming thought for those healthcare systems considered competent and efficient in providing safe and satisfactory care to patients.^7^ The frequency of psychosocial stress in Asian nurses is 95.5 % much higher than in Western countries and 21% found in Southern Indian nurses^8^, in Taiwan (2018) 66%^9^, in Nigeria 61% (2019)^10^, and in Pakistan 35.1% (2016).^11^

According to psychosocial theory, the stress-free engagement of nurses in their work is one of the vital components of achieving sustainable goals of the organization.^12^ Psychosocial stress is any type of hazard that might interfere with the mental process and compromise defense mechanisms.^13^ It is demonstrated in current research that psychosocial stressor, which is perceived and faced by nurses at the hospital, play a foremost part in arising conflicts and miscommunication among healthcare providers.^14^ It also considerably declines nurses’ cognitive, decision-making, and problem-solving ability, which ultimately impacts their performance profoundly.^15^

Many factors aggravate psychological and social stressors in the working area, such as conflicts among colleagues and task demands, unsafe working environment, poor support system, feeling insecure in the job, abuse and violence, random assignments, and unclear roles.^16^ Additionally, psychosocial stress has dire consequences because it can raise the probability of hospital incidents like failure to rescue, burnout of nurses, and job dissatisfaction.^17^ It is professed through a research study that psychosocial stress in the health care system impaired nurses’ health, resulting in adverse outcomes for patients.^18^ The present study aimed to assess the psychosocial stress of nurses and the relationship of psychosocial stress of nurses with a safety attitude toward nurses’ performance at Tertiary Care Hospital, Karachi.

### Theoretical framework:

### Generalized un-safety theory of stress

The generalized un-safety theory of stress (GUTS) illustrates a chief component of an unsafe context that might establish a stressful environment. In this the person overestimates stress and has less perception of safety. When a person has a consistently stressful environment, after a period, the perception of stress increases as compared to the perception of safety. It happened due to hyperarousal of worry about a safe environment. According to this model, the perception of stress predicts a stressful environment and compromising security. As a result, indicators of pathology enhanced their role in the development of psychological manifestation of disease a person’s energy is depleted due to prolonged exposure to stressors, and the person might manifest stress for a shorter time or even if it continues for a more extended period of time.^19^-^20^

## METHODS

The present analytical cross-sectional study was accomplished at Dr. Ruth KM Pfau Civil Hospital, Karachi, and Dow University Hospital Karachi for six months, from December 2020 to May 2021. Registered nurses with valid Pakistan Nursing and Midwifery Council (PN&MC) licenses were enrolled for the study. Nursing students and nurses who have a history of psychiatric illness or are on psychiatric medicine were excluded from the study. A non-probability purposive sampling method was used to approach the study participants.

### Ethical approval

It was granted by the Institutional Review Board of Dow University of Health Sciences, Karachi, Ref:1821/DUHS/Approval/2020/. Additionally, written approval was obtained from the Medical Superintendents of both study settings regarding the participation of registered nurses in the study. The study was conducted according to the Helsinki declaration of 2000.

The sample size was calculated through the WHO sample size software in health sciences determination. It was calculated by taking the frequency of psychosocial stress in nurses’ 21% in study^21^ with a significance level of 95% and a significant p value ≤0.05 considered for sample size calculation. The calculated sample size was 260 nurses of both genders.

Furthermore, the sample size was divided into two settings by considering 50% of the sample from each study setting. Written informed consent was taken from all participants, and the psychosocial stress instrument was explained explicitly to all participants before the data collection. Confidentiality and anonymity of the questionnaire responses were maintained. All the data was coded instead of names. Participants participated voluntarily.

An adopted and modified psychosocial stress questionnaire was applied. Regarding the validity and reliability of questionnaires, a pilot study was conducted among 10% of the same population of study that was not part of the study’s sample size. Kuder-Richardson 20 (KR-20) test was used. The alpha coefficient of the questionnaire was 0.84. and content validity index (CVI) of tool was 0.80.

In a current research study, the first part of the questionnaire consists of demographic variables. It comprises six questions: age, gender, marital status, highest education level, and working experience. The second part of the questionnaire contains psychosocial symptoms in which psychosocial stress was measured. It includes ten questions. Each question was interpreted on five points Likert scale (0 never, one rarely, 2 sometimes, 3 fairly often, and four very often). The following scales assessed the psychosocial stress: Poor Health Status (2.6 - 4.0), Good Health Status (1.3 - 2.6), and Best Health Status (0 - 1.33).

The third part of the questionnaire is related to the safety attitude questionnaire. It includes 32 questions, which consist of teamwork climate (1-6 questions), safety climate (7 – 13 questions), job satisfaction (15 – 19 questions), stress recognition (20 – 23 questions), perceptions of management (24 – 28 questions) and working conditions (29 – 32 questions). Each question was interpreted on a five-point Likert scale (disagree strongly- 1, disagree-2, neutral-3, agree slightly-4, agree with strongly-5).

### Statistical analysis

Data was entered and analyzed in SPSS version 21.0. The frequency of psychosocial stress of nurses was represented with percentages, and frequency and percentage were also used to show the relationship between the psychosocial stress of nurses and safety attitude towards nurses’ performances. Furthermore, Pearson’s correlation was used to establish the relationship between the psychosocial stress of nurses and different parameters of their safety attitude, and the Chi-square test was applied for the association between demographic factors of nurses with their psychosocial stress levels and associations between demographic factors of nurses and their safety attitude Score. A p-value was considered at ≤0.05 as significant.

## RESULTS

The demographic characteristics of the study participants are shown in Table-I. In the current study total of 260 participants were enrolled, and the percentage of female participants in the study was 163 (62.7%) whereas 97 (37.3%) were male and (65%) were married participant**s.** Most of the participants aged 119 (45.8%) between 36-45 years, 93 (35.8%) had four to eight years of working experience, and educational qualification of 115 (44.2%) was BSN Post RN. In addition, about 179 (68.8%) participants graduated from a public institution.

**Table-I:** Demographic Characteristics of Study Subjects (n=260).

S. No.	Variables	N	%
1	***Gender***		
	Male	97	37.3
	Female	163	62.7
2	***Age Category***		
	16-25 years	25	9.6
	26-35 years	100	38.5
	36-45 years	119	45.8
	46yrs and above	16	6.2
3	***Educational Degree***		
	General Diploma	50	19.2
	Midwifery	73	28.1
	Post RN	115	44.2
	Generic Nursing	22	8.5
4	***Marital Status***		
	Unmarried	64	24.6
	Married	169	65
	Divorced	21	8.1
	Widow	06	2.3
5	***Working Experiences***		
	1-4 years	50	19.2
	5-8 years	93	35.8
	9-12yrs	70	26.9
	13-16yrs	47	18.1
6	***Institutions***		
	Public	179	68.8
	Private	81	31.2

**Fig.1:**
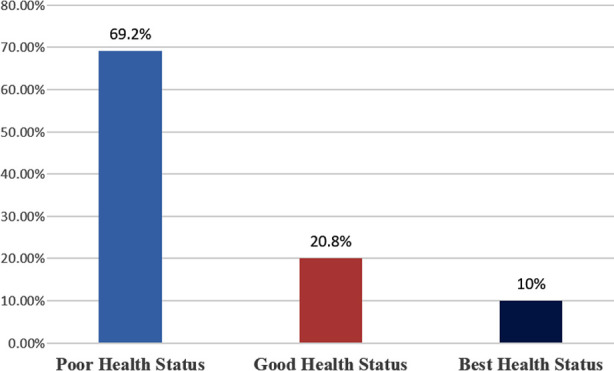
Frequency Distribution of psychosocial stress of nurses (n=260).

Frequency of psychosocial stress has been exhibited in Fig.1. The frequency of psychosocial stress in nurses which was found 180 (69.2%). The participants disclosed 54 (20.8%) with good health, and 26 (10%) participants reported their best health status.

The correlation between the psychosocial stress of nurses and different parameters of their safety attitude is shown in Table-II. Pearson correlation test was used to establish the correlation between the psychosocial stress of nurses and various parameters of safety attitude. It was found that among 06 parameters of safety attitude: 03 parameters are negatively correlated and statistically significant with psychosocial stress, namely: teamwork (r-0.13<0.002), job satisfaction (r-0.150<0.028), and perception of management (r-0.34<0.000). On the other hand, 02 parameters are significantly positively correlated with psychosocial stress, including Stress recognition (r0.166<0.0001) and working conditions (r0.099<0.01). Only the one parameter is not significantly correlated, which was safety climate (r 0.47<0.149).

**Table-II:** Correlation Between psychosocial stress of nurses and different parameters of their safety attitude (n=260)

	Parameters	Correlation value (r)	p-value
Psychosocial stress of nurses	Teamwork Climate	-0.13	0.0002*
	Safety Climate	0. 47	0.149
	Job Satisfaction	-0.150	0.028*
	Stress Recognition	0.166	0.0001*
	Perception of Management	-0.34	0.000
	Working Condition	0.099	0.01*

* Significance level at 0.05.

An association between the demographic factors of nurses and their psychosocial stress levels are shown in Table-III. The Chi-square test found a significant association between gender (P-value 0.0035), Age (P-value<0.000), and working experience (P-value<0.005) with psychosocial stress among participants. In contrast, marital status (P-value 0.716), education (P-value 0.246), and institute (P-value 0.879) have no significant association.

**Table-III:** Association between Demographic Factors of Nurses and their psychosocial stress level (n=260).

Demographic Factors of Nurses		The psychosocial stress level of Nurses Mean ± SD	p-value
Gender	Male	1.87±0.691	0.0035*
	Female	1.79±0.660	
Age	16-25 years	1.56±0.82	<0.000*
	26-35 years	1.85±0.67	
	36-45 years	1.86±0.629	
	46yrs and above	1.73±0.695	
Educational Degree	General Diploma	1.82±0.704	0.246
	Midwifery	1.92±0.558	
	Post RN	1.80±0.700	
	Generic Nursing	1.60±0.775	
Marital Status	Unmarried	1.87±0.686	0.716
	Married	1.81±0.673	
	Divorced	1.69±0.719	
	Widow	1.96±0.287	
Working Experience	1-4 years	1.98±0.652	<0.005*
	5-8 years	1.84±0.613	
	9-12years	1.74±0.793	
	13-16years	1.71±0.586	
Institutions	Public	1.82±0.655	0.879
	Private	1.81±0.713	

* Significance level at 0.05.

Associations between the demographic factors of nurses and their safety attitude score is shown in Table-IV. The Chi-Square test indicated that gender (P-value<0.000), marital status (P-value<0.0037), and institution (P-value <0.005) were significantly associated with safety attitude scores. At the same time, age (P-value 0.274) and educational degree (P-value 0.267) were not significantly associated.

**Table-IV:** Associations between Demographic Factors of Nurses and their Safety Attitude Score (n=260).

Demographic Factors of Nurses		Safety Attitude Score of Nurses Mean ± SD	p-value
Gender	Male	2.97±0.414	0.000
	Female	3.06±0.509	
Age	16-25 years	3.14±0.497	0.274
	26-35 years	2.99±0.414	
	36-45 years	3.02±0.507	
	46yrs and above	3.19±0.570	
Educational Degree	General Diploma	3.07±0.446	0.267
	Midwifery	3.03±0.436	
	Post RN	3.04±0.515	
	Generic Nursing	2.84±0.460	
Marital Status	Unmarried	3.03±0.533	0.0037*
	Married	2.99±0.450	
	Divorced	3.19±0.481	
	Widow	3.34±0.496	
Working Experience	1-4 years	3.02±0.420	0.515
	5-8 years	3.04±0.517	
	9-12 years	3.07±0.485	
	13-16 years	2.94±0.411	
Institutions	Public	3.04±0.487	<0.005*
	Private	3.00±0.456	

* Significance level at 0.05.

## DISCUSSION

The majority of nurses 69.2% (180) reported poor health, while 20.8% (54) had good health, and only 10% (26) of nurses reported their best health status. A systemic review has highlighted the psychosocial issues among registered nurses globally and locally.^22^

These study findings are congruent with a study conducted in Brazil by Dutra et al. (2021), which concluded that the burden of psychosocial stress is highest (71%) among nurses.^23^, and 52% of nurses are at risk of developing this issue reported by Okeafor CU et al. in Nigeria (2018).^24^ In contrast to our study, many studies have showed the psychosocial problems of registered nurses from different perspectives like psychosocial stress and workplace factors by Barrientos-Trigo S et al. study in 2018^4^, musculoskeletal pain and psychosocial stress in Estonia by Freimann T et al. in 2020.^25^

It was found that three parameters were negatively correlated and statistically significant with psychosocial stress, namely: teamwork (r-0.13<0.002), job satisfaction (r-0.150<0.028), and perception of management (r-0.34<0.000). As teamwork, job satisfaction, and perception of management increases, psychosocial stress decreases. It is also notable that two parameters were significantly positively correlated with psychosocial stress, including Stress recognition (r 0.166<0.0001) and working condition (r 0.099<0.01). Only the 01 parameter is not significantly correlated, which was safety climate working condition (r 0.47<0.149). Similar findings were supported by Dutra CK et al. in Brazil’s cross-sectional study.^24^ Opposite results were established in another study carried out in Brazil by Dorian GH that found only a positive correlation between safety attitude with job satisfaction, and a negative correlation was shown regarding the perception of management, safety climate, working conditions, safe behaviors, and job satisfaction.^22^

Chi-Square test indicated that gender (P-value<0.000), marital status (P-value<0.0037), and institution (P-value <0.005) were significantly associated with safety attitude scores while age (P-value 0.274), and educational degree (P-value 0.267) were not significantly associated. It was in line with a cross-sectional study by Sharmila et al. in India which showed that the age variable was not related to the psychosocial stress of nurses, and it was evaluated that working experience was associated with stress level. Further, it was concluded that the psychological, physical, and emotional domains were interlinked with each other.^26^ However, varied findings were unveiled by Islam MI et al. in Bangladesh. Study findings displayed that psychosocial stress among males was greater than in female participants and nurses whose ages were more than 40 years; the unmarried and working experience lies between 10 to 20 years.^27^

Additionally, it was highlighted that stress was related to other factors, which were different from our study, for instance: workload, conflict, role and responsibilities, and support system.^27^ Another cross-sectional study conducted by Karimi R et al. from Iran in 2014 portrayed that the psychosocial stress of nurses was associated with gender, marital stress, and educational degree, and it was also associated with long working hours, role conflict, ambiguous job responsibilities, and lack of organizational support for mental health.^28^

### Limitation of the study

It is analytical cross-sectional study with small sample size; hence, the generalization cannot be made. The study was accomplished in two public tertiary care hospitals. Therefore, the findings of study are not in line with private tertiary care hospitals. The results of the study are not comparable in rural setting hospitals as this study was employed in urban setting.

## CONCLUSION

The study’s findings conclude that most of study participants reported poor health, which significantly related to teamwork, job satisfaction and perception of management, and Stress recognition. However, the current study highlighted that a higher proportion is related to altered psychosocial stress in terms of poor health, which may ultimately decline the standards of nursing care.

### Authors Contributions:

**AW:** Conceived idea, designed the study and responsible for the integrity of the research.

**B:** Contributed to the acquisition, analysis, or interpretation of data.

**W:** Drafted and edited the manuscript.

All authors did a critical revision of the manuscript and approved it for publication.
